# Effectiveness of transcranial electrical stimulation combined with dual-task training in stroke, mild cognitive impairment and Parkinson’s disease: a systematic review and meta-analysis of randomized controlled trials

**DOI:** 10.3389/fnhum.2025.1688110

**Published:** 2026-01-16

**Authors:** Yutong Fu, Wenli Wang, Qianxi Yan, Chang Zhu, Siaw Chui Chai, Ponnusamy Subramaniam, Liqing Yao, Devinder Kaur Ajit Singh

**Affiliations:** 1 Department of Rehabilitation Medicine, The Second Affiliated Hospital of Kunming Medical University, Kunming, China; 2 Center for Healthy Ageing and Wellness (H-CARE), Faculty of Health Sciences, Universiti Kebangsaan Malaysia, Kuala Lumpur, Malaysia; 3 Centre for Rehabilitation and Special Needs Studies (iCaRehab), Faculty of Health Sciences, Universiti Kebangsaan Malaysia, Kuala Lumpur, Malaysia

**Keywords:** cognitive impairment, dual task training, neurological disorders, Parkinson’s disease, stroke, transcranial electrical stimulation

## Abstract

**Aim:**

In this systematic review and meta-analysis, we evaluated the effectiveness of transcranial direct current stimulation (tDCS) and transcranial alternating current stimulation (tACS) or transcranial random noise stimulation (tRNS) combined with dual task training (DTT) on physical and cognitive functions in adults with mild cognitive impairment (MCI), Parkinson’s disease (PD), and stroke disorders.

**Method:**

We conducted a systematic search of the Web of Science, MEDLINE, Cochrane Library, PubMed, and CINAHL databases for English-language literature on randomized clinical trials (RCTs) investigating the effects of tDCS, tACS, or tRNS combined with DTT in adults with MCI, PD, and stroke. The search covered studies from the inception of each database up to November 21, 2025. The initial screening of selected articles was conducted independently by two researchers (YLQ and WLW).

**Results:**

A total of twelve studies met the inclusion criteria, comprising individuals with stroke (n = 4), MCI (n = 3), and PD (n = 5). Meta-analysis revealed that active tDCS+DTT yielded no significant overall improvement in global cognition (Montreal Cognitive Assessment (MoCA): SMD = 0.09, 95% CI [−0.49, 0.66], *p* = 0.77, I^2^ = 72%). A large, highly homogeneous benefit was observed for executive function (TMT-B: SMD = −1.33, 95% CI [−2.39, −0.27], *p* = 0.01), driven exclusively by the MCI subgroup (SMD = −2.35, 95% CI [−3.20, −1.51], I^2^ = 0%). Timed Up and Go cognitive-motor dual-task (TUG CMDT) cadence improved overall (SMD = 0.58, 95% CI [0.09, 1.08], *p* = 0.02, I^2^ = 39%) in both MCI and stroke subgroups. TUG motor dual task (MDT) speed improved modestly (SMD = 0.42, 95% CI [0.02, 0.83], *p* = 0.04, I^2^ = 34%), and CMDT speed showed a strong trend (SMD = −0.49, *p* = 0.09), only significant in stroke (SMD = −1.42, *p* = 0.002). However, this generalized finding must be nuanced by specific efficacy observed in individual PD studies, which reported significant gains in force-tremor decoupling, postural stability, and CMDT accuracy.

**Conclusion:**

The meta-analysis suggests that the effects of tDCS combined with DTT are remarkable in certain populations and for specific outcomes. While substantial improvements are confirmed for executive function and dual-task gait in MCI and stroke, the overall limited efficacy in PD highlights the critical influence of heterogeneity and intervention specificity. Future research should prioritize disease-specific electrode montages and the integration of tACS or tRNS to optimize outcomes across diverse neurological populations.

## 1 Introduction

Neurological disorders have become the leading cause of the global disease burden. In 2021, more than 3 billion people worldwide were affected by neurological disorders, with the total disability-adjusted life years (DALYs) attributable to these disorders increasing by 18% compared to 1990 (Steinmetz, et al., 2024). Over 80% of deaths and health losses caused by neurological disorders occurred in low- and middle-income countries, where significant disparities in treatment outcomes exist. Furthermore, in 2021, neurological disorders impaired the quality of life of 443 million people, making them the primary contributor to the global disease burden, surpassing cardiovascular diseases (Martin et al., 2024). Neurological disorders encompass a wide range of conditions affecting the brain and nerves, including stroke, Parkinson’s disease (PD), Alzheimer’s disease, epilepsy, multiple sclerosis, and traumatic brain injury. These conditions often impair cognitive (Zegarra-Valdivia et al., 2025), motor (Wu et al., 2025), sensory (Hoh and Semrau, 2025), socioemotional (Zegarra-Valdivia et al., 2025) functions, and behavior (Blokland et al., 2023).

Non-invasive brain stimulation (NIBS), such as transcranial direct current stimulation (tDCS), transcranial alternating current stimulation (tACS), or transcranial random noise stimulation (tRNS), is an effective adjunct to conventional training to improve motor and cognitive function (Andre et al., 2016). Transcranial electrical stimulation (tES) is a NIBS technique of neuromodulation to generate specific changes in cortical excitability (Sánchez-Kuhn et al., 2017). tES involves placing electrode pads on the surface of the skull in specific configurations to deliver low-intensity currents (1 to 2 mA) to the cerebral cortex, thereby modulating brain activity and the corresponding human behaviors (Brown et al., 2022).

tDCS has shown significant potential in the treatment of various neurological disorders in recent years. It has been investigated for its applications in epilepsy (Ding et al., 2025), Alzheimer’s disease (Cornea et al., 2025), PD (Giustiniani et al., 2025), stroke (Qin et al., 2025), and attention disorders (Steen-García et al., 2024). The mechanisms of action involve modulating brain activity and influencing human behavior by delivering low-intensity electrical currents to the cerebral cortex. It is based on the principles of neuroplasticity, which refer to the brain’s ability to change and adapt in response to experience, learning, or injury (Ehsani et al., 2025; Yao et al., 2025). tDCS can induce neuroplastic changes by influencing neuronal activity and synaptic plasticity, and by promoting the formation of new neural connections (Chan et al., 2021). tDCS has demonstrated safety and efficacy in enhancing motor and cognitive function across neurological conditions, including stroke (Corominas-Teruel et al., 2023), PD (Schabrun et al., 2016), and Alzheimer’s disease (Duan and Zhang, 2024). While its mechanisms remain under investigation, tDCS shows promise for treating motor, cognitive, and mood symptoms in neurological disorders, including post-stroke depression (Oh et al., 2022; Ding et al., 2025).

tACS is a non-invasive technique that applies weak electrical currents (1–2 mA) at specific frequencies to modulate brain oscillations through electrodes placed on the scalp. By delivering various stimulation patterns (alpha, beta, gamma, and theta), tACS influences neural synchronization and desynchronization, showing therapeutic potential across neurological conditions. Research demonstrates tACS efficacy for cognitive and motor recovery in stroke survivors (Han et al., 2025; Takeuchi and Izumi, 2021) and for improving motor and cognitive symptoms in PD by modulating abnormal beta and gamma oscillations (Guerra et al., 2020; Morelli and Summers, 2023). Similarly, tRNS appears to function through stochastic resonance, a phenomenon in which optimal noise enhances weak-signal detection (van der Groen et al., 2022), with preliminary clinical trials showing improved upper limb function in stroke survivors (Sethi et al., 2023; van der Groen and Wenderoth, 2016).

Dual-task training (DTT) has also emerged as a promising intervention for enhancing functional recovery in neurological conditions. Poor dual-task performance negatively impacts daily activities (Lemke et al., 2019), making DTT increasingly important for motor and cognitive rehabilitation (Tuena et al., 2023). DTT refers to the simultaneous execution of two tasks, which may involve two motor tasks (motor–motor) or a combination of a cognitive and a motor task (CMDT; Zhang et al., 2024b). Research demonstrates DTT efficacy across multiple neurological populations, including PD (Gaßner et al., 2022), Alzheimer’s disease (Longhurst et al., 2023), stroke (Zhang et al., 2022), and older adults (Ali et al., 2022). For stroke survivors specifically, consistent DTT improves walking speed, balance, and cognitive function and reduces fall risk (Zhang et al., 2022). Combined walking and cognitive training enhances flexibility (Peristeri et al., 2021) and self-care abilities (Trombini-Souza et al., 2020), with both CMDT and motor dual-task (MDT) walking requiring greater attentional resources than single-task walking (Hillel et al., 2019).

The central-peripheral-central closed-loop rehabilitation theory suggests that combining dual-task (DT) gait training with transcranial electrical stimulation (tES) may enhance rehabilitation outcomes, with evidence indicating that tES effects are state-dependent (de Paz et al., 2019; Wang et al., 2025). Applying tES combined with tasks targeting the same brain networks may augment cortical excitability and neuroplasticity, enhancing therapeutic effects (Wiltshire and Watkins, 2020). This synergistic approach could maximize the impact of individual interventions. However, significant knowledge gaps remain regarding state-dependency effects on DT costs during walking and whether combining tES with behavioral tasks will positively affect motor-cognitive interference during DT performance, with further questions about the polarity and timing-dependent effects of tES in explicit motor learning when used in combination therapies.

To our knowledge, no systematic review and meta-analysis has been conducted to evaluate the combined approach, which could provide alternative rehabilitation strategies for improving mobility and cognition in people with MCI, PD, and stroke disorders. Therefore, the aim of this systematic review and meta-analysis was to synthesize the available evidence on the effects of concurrent tES and DTT on physical and cognitive outcomes in these three major neurological disorders.

## 2 Materials and methods

### 2.1 Data collection

This systematic review was conducted in accordance with the Preferred Reporting Items for Systematic Reviews and Meta-Analyses (PRISMA) guidelines and recommendations by Page et al. (2021). The selected articles were screened preliminarily by two independent researchers (YLQ, WLW) who screened them individually. If the literature and abstract met the inclusion criteria, the full text was then reviewed, and specific analyses of the experiments were conducted to determine final inclusion. Any discrepancies were resolved through discussion with a third researcher (FYT).

We searched Web of Science Core Collection, Medline (via Ovid), Embase (via Ovid), PubMed, and CINAHL Complete from inception to 21 November 2025. The search strategy combined controlled vocabulary (MeSH or Emtree) and free-text terms and was restricted to randomized controlled trials (RCTs). The final Ovid-Medline string is shown below; it was adapted for the other databases.

Population (stroke OR MCI OR PD).

1. Stroke/ OR stroke*.tw,kf. OR poststroke.tw,kf. OR post-stroke.tw,kf. OR cerebrovascular accident*.tw,kf. OR cerebral infarction/ OR brain infarction*.tw,kf. OR cerebral hemorrhage/ OR intracerebral hemorrhage.tw,kf. OR ischemic stroke.tw,kf. OR hemorrhagic stroke.tw,kf. OR brain ischemia.tw,kf.
2. Parkinson disease/ OR parkinson*.tw,kf. OR Parkinson’s disease.tw,kf. OR PD.tw,kf.
3. Mild cognitive impairment/ OR mild cognitive impairment.tw,kf. OR MCI.tw,kf.
4. 1 OR 2 OR 3.
  Intervention (tES with dual-task training).
5. Transcranial direct current stimulation/ OR transcranial electrical stimulation/ OR transcranial alternating current stimulation/ OR transcranial random noise stimulation/ OR tDCS.tw,kf. OR transcranial direct current stimulation.tw,kf. OR transcranial electrical stimulation.tw,kf. OR tACS.tw,kf. OR tRNS.tw,kf. OR transcranial alternating current stimulation.tw,kf. OR transcranial random noise stimulation.tw,kf.
6. Dual task/ OR dual task*.tw,kf. OR dual-task*.tw,kf. OR cognitive motor interference.tw,kf. OR divided attention.tw,kf. OR attention-demanding task*.tw,kf. OR multitasking.tw,kf. OR concurrent task.tw,kf. OR interference cost.tw,kf. OR cognitive motor interference.tw,kf. OR combined training.tw,kf. OR combined intervention.tw,kf.
7. 5 AND 6.

Study design (RCT).

1. Randomized controlled trial.pt. OR controlled clinical trial.pt. OR randomi#ed.ab. OR placebo.ab. OR randomly.ab. OR trial.ab. OR groups.ab.
2. 4 AND 7 AND 8.

In Pubmed style (“Stroke”[Mesh] OR “Mild Cognitive Impairment”[Mesh] OR “Parkinson Disease”[Mesh]) AND (“Transcranial Direct Current Stimulation”[Mesh] OR “Transcranial Electrical Stimulation”[Mesh] OR tDCS[tiab] OR tACS[tiab] OR tRNS[tiab]) AND (“(“Dual Task”[Mesh] OR dual-task*[tiab] OR “cognitive motor interference”[tiab] OR “divided attention”[tiab] OR “attention-demanding task*”[tiab] OR multitasking[tiab] OR “concurrent task*”[tiab] OR “task prioritization”[tiab] OR “interference cost*”[tiab] OR “combined training”[tiab] OR “motor cognitive”[tiab])) AND (“Randomized Controlled Trial”[pt] OR random*[tiab]).

We selected only articles and reviews in English and excluded other document types, such as letters, commentaries, and meeting abstracts. These terms were used in combination with “AND” and “OR.”

### 2.2 Data import and deduplication

**Figure 1:**
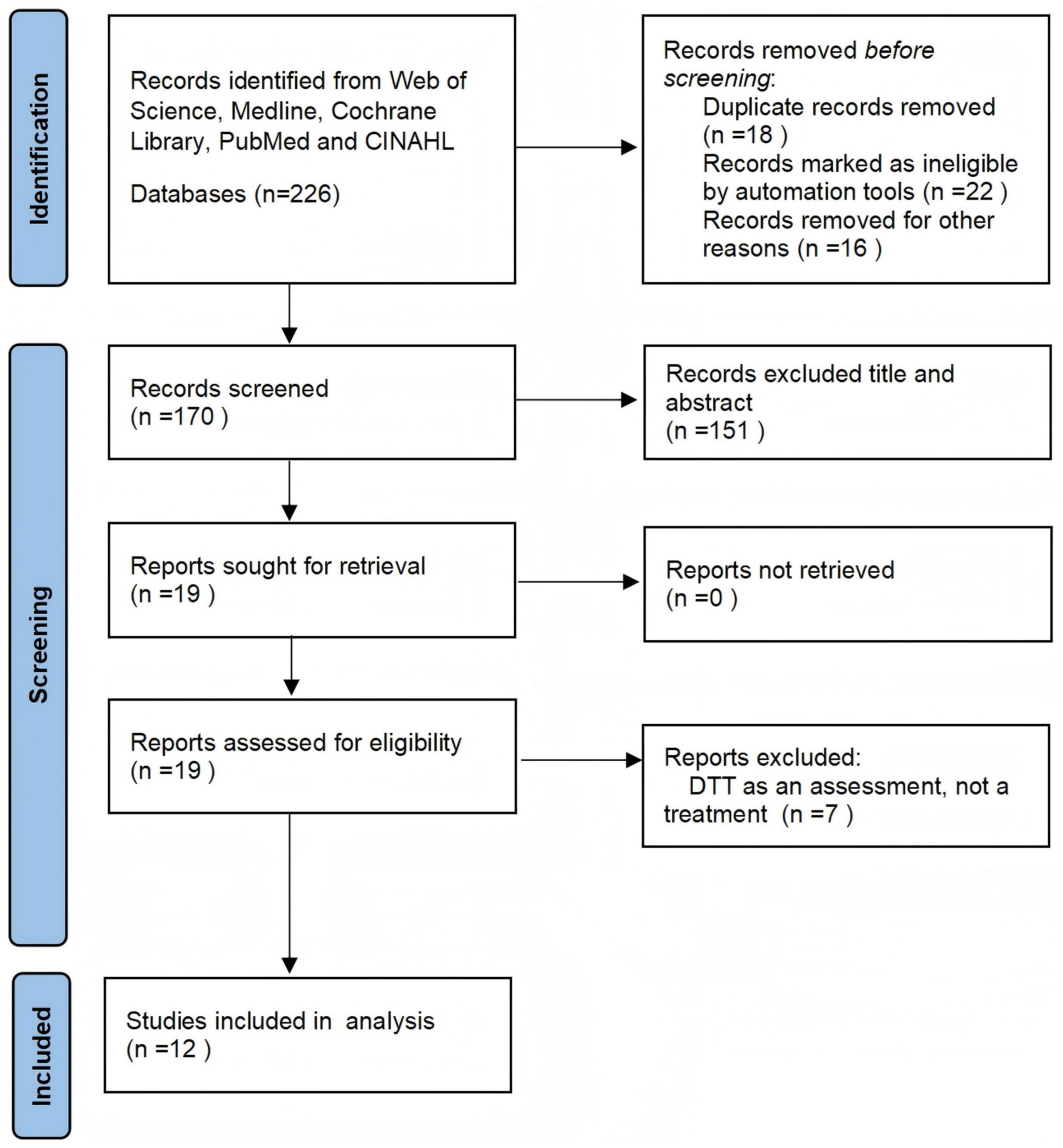
Flow chart of the effects of tES-related RCT research on survivors of neurological disorders on cognitive and physical functions.

In this study, the publication type was restricted to original research articles. We downloaded all articles and then read the titles, abstracts, and full texts of the included papers to identify the final available studies. Deduplication was performed using EndNote software, which automatically identifies and removes duplicate records based on title, abstract, and author information. Furthermore, the automated results underwent a manual validation by two independent researchers (YLQ and WLW) to guarantee the exclusion of all duplicate entries. Any discrepancies were resolved through discussion with a third researcher. Inclusion criteria are as follows: (1) Population: adults with stroke/MCI/PD; (2) Intervention type: tES combined with DTT; (3) The control group received sham stimulation with DTT or with no stimulation, only DTT; (4) Study designs: Randomized controlled trials (RCTs); (5) Moca and TUG DT scales as the primary outcomes and any cognitive or motor scales or gait analysis as the secondary outcomes. Exclusion criteria are as follows: (1) Observational studies, reviews, and case reports. Case reports; (2) Letters (3) Studies focusing on other types of brain stimulation (magnetic stimulation) or interventions not involving tES were excluded. Furthermore, the language was restricted to English, while the literature publication period spanned from database inception to November 21, 2025. The PubMed search and analysis flowchart is presented in Figure 1.

### 2.3 Data extraction

Included studies were independently assessed by two experienced reviewers based on the above inclusion/exclusion criteria. Extracted data and descriptive information package, including general characteristics, such as the first author, year of publication, age, number of subjects, patient type, and stimulation parameters, as shown in Tables 1–3. Outcomes are detailed in Table 4; these include any cognitive outcomes measured using the Montreal Cognitive Assessment (MoCA) and other related cognitive scales. Dual- or single-task walking tests were evaluated using gait parameters, including walking speed, cadence, step length, step time, and others. Upper- and lower-limb functional measures, muscle strength, Activities of Daily Living (ADL), safety assessments, such as adverse events or side effects related to the intervention, and other biomarkers are presented in Table 4. Any disagreements were resolved through discussion and consultation with a third researcher.

**Table 1:** Characteristics of MCI studies.

First author (publication, year, country)	Title	Sample size (male, female), age, (years±SD)	Stroke etiology,location, affected hemisphere	Time onset	Design,intensity	Technique,time	Period	Electrodes location anode	Cathode	Follow up	Other therapy
Xu et al. (2023), China	Effects of Tai Chi combined with tDCS on cognitive function in patients with MCI: a randomized controlled trial	93,31 male,62 female, 60.05 ± 8.63	-	-	2 mA Anodal +sham	tDCS,20 min	12 weeks,24 sessions	9 mm^2^, F4	Left supraorbital	no	Tai Chi
Lau et al. (2024), China, Taiwan	Can transcranial direct current stimulation combined with interactive computerized cognitive training boost cognition and gait performance in older adults with mild cognitive impairment? a randomized controlled trial	21,14 female,7 male,70.97 ± 12.98	-	-	2 mA Anodal + sham	tDCS,20 min	3/week for 5 weeks,15 training sessions	35 cm^2^, left DLPFC	Right supraorbital	no	Computerized Cognitive Training
Liao et al. (2021), China	Combining Transcranial Direct Current Stimulation with Tai Chi to Improve Dual-Task Gait Performance in Older Adults with Mild Cognitive Impairment: A Randomized Controlled Trial	20,7 male,13 female, 72.85 ± 4.36	20 left	-	2 mA Anodal +sham	tDCS,20 min	36 sessions over 12 weeks.	35 cm^2^, left DLPFC	Right supraorbital	no	Tai Chi

**Table 2:** Characteristics of PD studies.

First author (publication, year, country)	Title	Sample size (male, female), age, (years±SD)	Stroke etiology,location, affected hemisphere	Time onset	Design,intensity	Technique,time	Period	Electrodes location anode	Cathode	Follow up	Other therapy
Schabrun et al. (2016), Australia	Transcranial Direct Current Stimulation to Enhance Dual-Task Gait Training in Parkinson’s Disease: A Pilot RCT	16, 10 male, 6 female, 67.5 ± 8.51	-,	5.75 ± 4.16 years	2 mA Anodal +sham	tDCS,20 min	3 sessions per week for 3 weeks	35 cm^2^ left primary motor cortex (M1)	35 cm^2^ contralateral supra-orbital	12 week	
Wong et al. (2024), China, Taiwan	Effects of DLPFC tDCS Followed by Treadmill Training on Dual-Task Gait and Cortical Excitability in Parkinson’s Disease: A Randomized Controlled Trial	34, 14 male, 20 female, 67.45 ± 6.31	-	6.7 ± 5.06 years	2 mA Anodal +sham	tDCS,20 min	2 to 3 sessions per week in 5 weeks for a total of 12 sessions	35 cm^2^, left DLPFC	Right supraorbital	no	Treadmill training
Pisano et al. (2024), Italy	Cerebellar tDCS combined with an augmented reality treadmill for freezing of gait in Parkinson’s disease: a randomized controlled trial	17, 7 male, 10 female 68.51 ± 9.08	-	-	2 mA Anodal +sham	tDCS,20 min	5/week, 2 wks, 10 sessions	35 cm^2^, cerebellar	Right arm	4 week	Treadmill
de Almeida et al. (2024), Brazil	Combining Transcranial Direct Current Stimulation with Exercise to Improve Mobility, Stability, and Tremor Management in 25 Individuals with Parkinson’s Disease	16, 10 males, 6 females, 66.81 ± 3.36	-	-	2 mA Anodal +sham	tDCS,20 min	5/week, 2 wks, 10 sessions	35 cm^2^, CZ	FP2	-	Dual-task

**Table 3:** Characteristics of stroke studies.

First author (publication, year, country)	Title	Sample size (male, female), age, (years±SD)	Stroke etiology, location, affected hemisphere	Time onset	Design,intensity	Technique,time	Period	Electrodes location anode	Cathode	Follow up	Other therapy
Aneksan et al. (2022), Thailand	Five-Session Dual-Transcranial Direct Current Stimulation with Task-Specific Training Does Not Improve Gait and Lower Limb Performance Over Training Alone in Subacute Stroke: A Pilot Randomized Controlled Trial	25,17 male,8 female, 54.36 ± 12.61	12 left, 13 right	92.11 ± 43.49 days	2 mA dual +sham	tDCS,20 min	5 consecutive daily sessions.	35 cm2 Anode:lesioned hemisphere M1	Cathodal:nonlesioned hemisphere M1	1 month	Physical therapy
Wong et al. (2022), China, Taiwan	Comparing different montages of transcranial direct current stimulation on dual-task walking and cortical activity in chronic stroke: double-blinded randomized controlled trial	24,19 male, 5 female, 11 left, 13 right, 55 (19.2)	10 ischaemic, 14 haemorrhagic	64.2 (73.0)	2 mA duall+sham	tDCS,20 min	one session	35 cm2 Anode: M1 position of the ipsilesional hemisphere, C3/C4	Contralateral supraorbital ridge	no	no
Wong et al. (2023), China, Taiwan	Effects of transcranial direct current stimulation followed by treadmill training on dual-task walking and cortical activity in chronic stroke: a double-blinded randomized controlled trial	28,22 male, 6 female, 9 left 19 right 62.3 (9.4–11.3)	19 ischaemic, 9 haemorrhagic	63.5 (38.7–57.1)	2 mA Cathodal +sham	tDCS,50 min	3 sessions per week for 4 weeks	35 cm2 Anode:contralateral supraorbital ridge	35 cm^2^: M1 position of the ipsilesional hemisphere, C3/C4	no	Treadmill training
Zhang et al. (2024a), China	Impact of transcranial direct current stimulation combined with motor-cognitive intervention on post-stroke cognitive impairment	60, 34 male, 26 female, 56.1 ± 5.81	-	8.32 ± 2.88 weeks	2 mA Anodal + no tdcs only physical therapy	tDCS,20 min	5 times a week, 4 weeks	35 cm2,left DLPFC	Right supraorbital	no	Motor-cognitive intervention
Lee and Cha (2022), Korea	The effect of clinical application of transcranial direct current stimulation combined with non-immersive virtual reality rehabilitation in stroke patients	20, 13 males, 7 females, 66.25 ± 6.36	15 ischemic, 5 hemorhage, 5 right, 15 left	3.94 ± 1.53 (months)	2 mA Anodal +sham	tDCS,20 min	5/week, 4 wks, 20 sessions	Affected M1 (C3/C4)	Contralateral orbit	-	VR

**Table 4:** Outcomes.

Type	Outcomes	Cognition	Gait/walking	Motor function	ADL	Electrophysiology	Safety assessment	Grade I II III IV
Stroke	Wong et al. (2022), China		GAITRite system Parameter; ST walking; DT walking;					I&II
	Wong et al. (2023) China		GAITRite system Parameter; ST walking; DT walking;			MEP		I&II
	Aneksan et al., (2022), Thailand		Force distribution measurement; Gait parameters; TUG	muscle parameters;				I&II
	Lee and Cha (2022), Korea	TMT, Stroop			BBT, JTHFT			II&III
	Zhang et al. (2024a), China	MoCA; LOTCA;					mild adverse reactions 2.2%	
MCI	Liao et al. (2021), China	MoCA; Chinese Version of the Verbal Learning Test: episodic memory Visual Working Memory; Tower of London Task; Trail Making Test; Stroop	GAITRite system Parameter; ST walking; DT walking;					I&II
	Lau et al. (2024), China	MoCA; TMT; Tower of London Task; CVVLT; Episodic memory; N-back;	GAITRite system Parameter; ST walking; DT walking;					I&II
	Xu et al. (2023), China	MoCA; Chinese Wechsler Memory Scale-Revised Memory Quotient: memory; Stroop Auditory learning test: Rey-Osterrieth complex figure: Test of Attentional Performance						I&II
PD	Wong et al. (2024), China	TMT	GAITRite system Parameter; ST walking; DT walking; TUGcognitive DT: verbally serial subtracting by 3 motor DT: walking with a bottle of water with the non-affected hand; Parameter: Speed; Cadence; Stride length; DTC of speed Stride time;		PDQ-39	Corticospinal Excitability RMT MEP		I&II
	Schabrun et al. (2016), Australia	Visuomotor speed and procedural learning: Serial Reaction Time Task	GAITRite system; gait parameters; DT walking; TUG	hand and elbow function, MDS- UPDRS III, Hoehn-Yahr				I&II
	Pisano et al. (2024), Italy	MoCA, MMSE	6 MWT, TUG, FoG-Q,’ C-Mill scores	Mini-BEST, UPDRS-III=Unified Parkinson’s Disease Rating Scale-Part III	BI, IADL, PDQ-8			III&IV
	de Almeida et al. (2024), Brazil		TUG, Functional Limit Test	Force and Acceleration Coherence, Accelerometry, Force, Hand Grip				III&IV

MoCA, Montreal Cognitive Assessment; MMSE, Mini-mental state examination; FTSTS, Five times sit to stand; TUG, Timed up and go; MEP, Motor-evoked potential; BBT, BoxandBlockTest; JTHFT, Jebsen-Taylor HandFunctionTest, ST, Strooptest; TMT: TrailMakingTest; CVVLT, Chinese Version of the Verbal Learning Test; Stroop, Stroop Color and Word Test.

### 2.4 Quality evaluation

**Figure 2:**
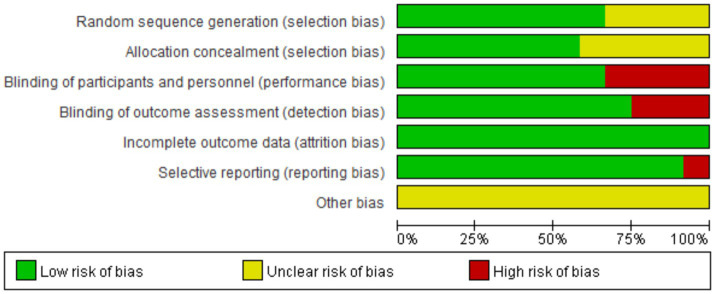
Risk of bias graph.

**Figure 3:**
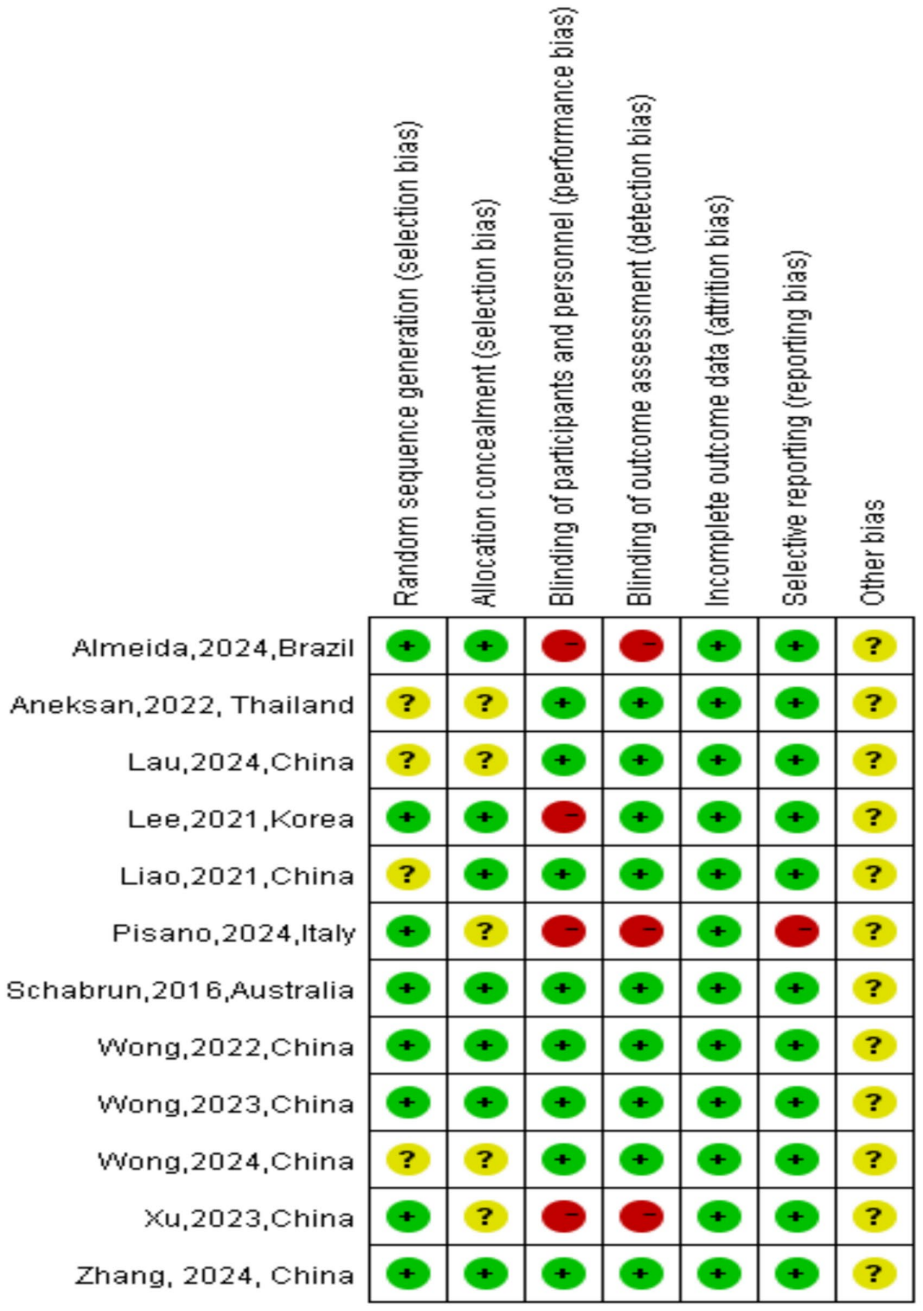
Risk of bias summary.

Two researchers used the Cochrane Manual Version 5.1.0 Risk of Bias Assessment Tool to independently assess risk assessment. The methodological standards for assessment are as follows (Higgins et al., 2011): The risk of bias assessment tool includes seven items: ① Generation of random sequences; ② Allocation concealment; ③Blinding of patients and practitioners; ④ Blinding in outcome assessment; ⑤ Incomplete data reporting; ⑥ Selective reporting; ⑦ Other sources of bias. If all the criteria were met, the study is considered to have the lowest possibility of bias. If the criteria are partially met, the study was categorized as a moderate possibility of bias. Finally, if one or more of the criteria are completely unsatisfied, the study was labeled as having high potential for bias, as shown in Table 5 and Figures 2, 3.

**Table 5:** PEDro scales.

Item	Stroke					MCI			PD			
	Zhang et al. (2024b), China	Wong et al. (2022), China	Wong et al. (2023), China	Aneksan et al. (2022), Thailand	Lee and Cha (2022), Korea	Liao et al. (2021), China	Lau et al. (2024), China,	Xu et al. (2023), China	Wong et al. (2024), China	Schabrun et al. (2016), Australia	Pisano et al. (2024), Italy	de Almeida et al. (2024), Brazil
Eligibility criteria	Yes	Yes	Yes	Yes	Yes	Yes	Yes	Yes	Yes	Yes	Yes	Yes
Random allocation	Yes	Yes	Yes	Yes	Yes	Yes	Yes	Yes	Yes	Yes	Yes	Yes
Concealed allocation	No	No	No	No	No	Yes	No	No	No	Yes	Yes	Yes
Baseline comparability	Yes	Yes	Yes	Yes	Yes	Yes	Yes	Yes	No	Yes	Yes	Yes
Blinding of subjects	Yes	Yes	Yes	Yes	Yes	Yes	Yes	No	Yes	Yes	Yes	Yes
Blinding of therapists	No	No	No	No	No	No	No	No	No	No	No	No
Blinding of assessors	Yes	Yes	Yes	Yes	Yes	Yes	Yes	No	Yes	Yes	No	No
Morethan 85% follow-up	Yes	Yes	Yes	Yes	Yes	Yes	Yes	Yes	Yes	Yes	Yes	Yes
Between-group statistical comparison	Yes	Yes	Yes	Yes	Yes	Yes	Yes	Yes	Yes	Yes	Yes	Yes
Measures of variability/precision	Yes	Yes	No	Yes	Yes	Yes	Yes	Yes	Yes	Yes	Yes	Yes
Total	8	8	7	8	8	9	8	6	7	9	8	8

## 3 Results

### 3.1 Intervention parameters and protocol

Among the 12 RCTs, 9 studies (75%) employed anodal tDCS stimulation protocols (Schabrun et al., 2016; Liao et al., 2021; Lau et al., 2024; Xu et al., 2023; Wong et al., 2024; Zhang et al., 2024a; Lee and Cha, 2022; Pisano et al., 2024; de Almeida et al., 2024), 1 studies (8.33%) utilized cathodal stimulation (Wong et al., 2023), and 2 studies (16.67%) implemented dual stimulation (Aneksan et al., 2022; Wong et al., 2022). All studies (100%) administered a current intensity of 2 mA during the tDCS interventions.

As for the electrode configuration, two electrode sizes were used across the included studies: 35 cm^2^ electrodes in 10 studies (83.33%; Schabrun et al., 2016; Liao et al., 2021; Lau et al., 2024; Wong et al., 2024; Zhang et al., 2024a; Pisano et al., 2024; de Almeida et al., 2024; Aneksan et al., 2022; Wong et al., 2022; Wong et al., 2023), and 9 cm^2^ electrodes in 1 study (8.33%; Xu et al., 2023). For anode placement, 4 studies (33.33%) positioned the anode over the primary motor cortex (M1; Schabrun et al., 2016; Lee and Cha, 2022; Aneksan et al., 2022; Wong et al., 2022), 4 studies (33.33%) targeted the left dorsolateral prefrontal cortex (DLPFC; Liao et al., 2021; Lau et al., 2024; Wong et al., 2024; Zhang et al., 2024a), and 2 studies (16.67%) placed the anode over the supraorbital area or F4 (Xu et al., 2023; Wong et al., 2023), 1 study (8.33%) placed on the Cz (de Almeida et al., 2024), one study (8.33%) on cerebellar (Pisano et al., 2024). For cathode placement, seven studies (77.8%) positioned the cathode over the supraorbital ridge/FP2/orbit (Schabrun et al., 2016; Liao et al., 2021; Lau et al., 2024; Xu et al., 2023; Wong et al., 2024; Zhang et al., 2024a; Lee and Cha, 2022; de Almeida et al., 2024; Wong et al., 2022), while two studies (22.2%) placed it over M1 (Aneksan et al., 2022; Wong et al., 2023) and 1 study was placed on the right arm (Pisano et al., 2024).

The majority of studies (11 studies, 91.67%) administered tDCS for 20 min per session (Schabrun et al., 2016; Liao et al., 2021; Lau et al., 2024; Xu et al., 2023; Wong et al., 2024; Zhang et al., 2024a; Lee and Cha, 2022; Pisano et al., 2024; de Almeida et al., 2024; Aneksan et al., 2022; Wong et al., 2022), with only 1 study (8.33%) implementing a longer duration of 50 min (Wong et al., 2023). The stimulation frequency varied from 1 to 5 sessions per week, with durations of 1, 3, 4, 5, or 12 weeks. Total treatment sessions across studies ranged from 1 to 36 sessions.

In most studies (8 studies, 77.8%), follow-up assessments after the completion of the intervention period were not conducted (Schabrun et al., 2016; Liao et al., 2021; Lau et al., 2024; Xu et al., 2023; Wong et al., 2024; Pisano et al., 2024; Wong et al., 2022; Wong et al., 2023). Of the studies that did include follow-up evaluations, two studies (16.67%) assessed outcomes at 1 month post-intervention (Pisano et al., 2024; Aneksan et al., 2022), and one study (8.33%) conducted assessments at 12 weeks post-intervention (Schabrun et al., 2016).

All experimental studies combined tDCS with conventional rehabilitation approaches, primarily physical and occupational therapy. Three studies (25%) incorporated treadmill training alongside tDCS intervention (Wong et al., 2024; Pisano et al., 2024; Wong et al., 2023), 1 study (11.1%) implemented computer-aided therapy in conjunction with tDCS (Lau et al., 2024), 1 (11.1%) study used VR (Lee and Cha, 2022). These innovative combinations aimed to explore potential synergistic effects on cognitive and motor functions among patients with stroke, PD, and MCI.

A notable methodological limitation observed across studies was the difficulty in blinding therapists to the treatment condition, likely due to the operational requirements of tDCS device administration. This limitation potentially introduces bias in the assessment and interpretation of outcomes.

### 3.2 Methodological quality assessment

The methodological quality of the nine included RCTs was assessed using the PEDro Scale and the Cochrane Collaboration tool for assessing risk of bias. PEDro Scale scores ranged from 1 to 10 (two studies scoring 7 (Wong et al., 2024; Wong et al., 2023), 7 scoring 8 (Lau et al., 2024; Zhang et al., 2024a; Lee and Cha, 2022; Pisano et al., 2024; de Almeida et al., 2024; Aneksan et al., 2022; Wong et al., 2022), 2 scoring 9 (Schabrun et al., 2016; Liao et al., 2021), indicating high methodological quality across all studies. According to the Cochrane risk-of-bias tool, all studies established appropriate eligibility criteria and demonstrated baseline comparability between groups. Most studies (11/12) successfully blinded participants and (9/12) outcome assessors, though a universal limitation was the inability to blind therapists due to the operational requirements of tDCS device administration.

Furthermore, in all studies, strong participant retention was maintained with none losing more than 20% during follow-up, and all conducted appropriate inter-group comparisons with adequate measures of variability. However, only two studies performed intention-to-treat analysis (Xu et al., 2023; Wong et al., 2023), representing a notable methodological limitation. Random sequence generation was clearly reported in all studies (Schabrun et al., 2016; Liao et al., 2021; Lau et al., 2024; Xu et al., 2023; Wong et al., 2024; Zhang et al., 2024a; Lee and Cha, 2022; Pisano et al., 2024; de Almeida et al., 2024; Aneksan et al., 2022; Wong et al., 2022; Wong et al., 2023) and allocation concealment was clearly reported in 4 studies (Schabrun et al., 2016; Liao et al., 2021; Pisano et al., 2024; de Almeida et al., 2024). Despite these limitations, the overall methodological quality of the included studies was high, with consistent strengths in participant blinding, assessor blinding, participant retention, and statistical reporting practices.

## 4 Meta-analysis results

### 4.1 Primary cognitive (Moca) outcome

**Figure 4:**
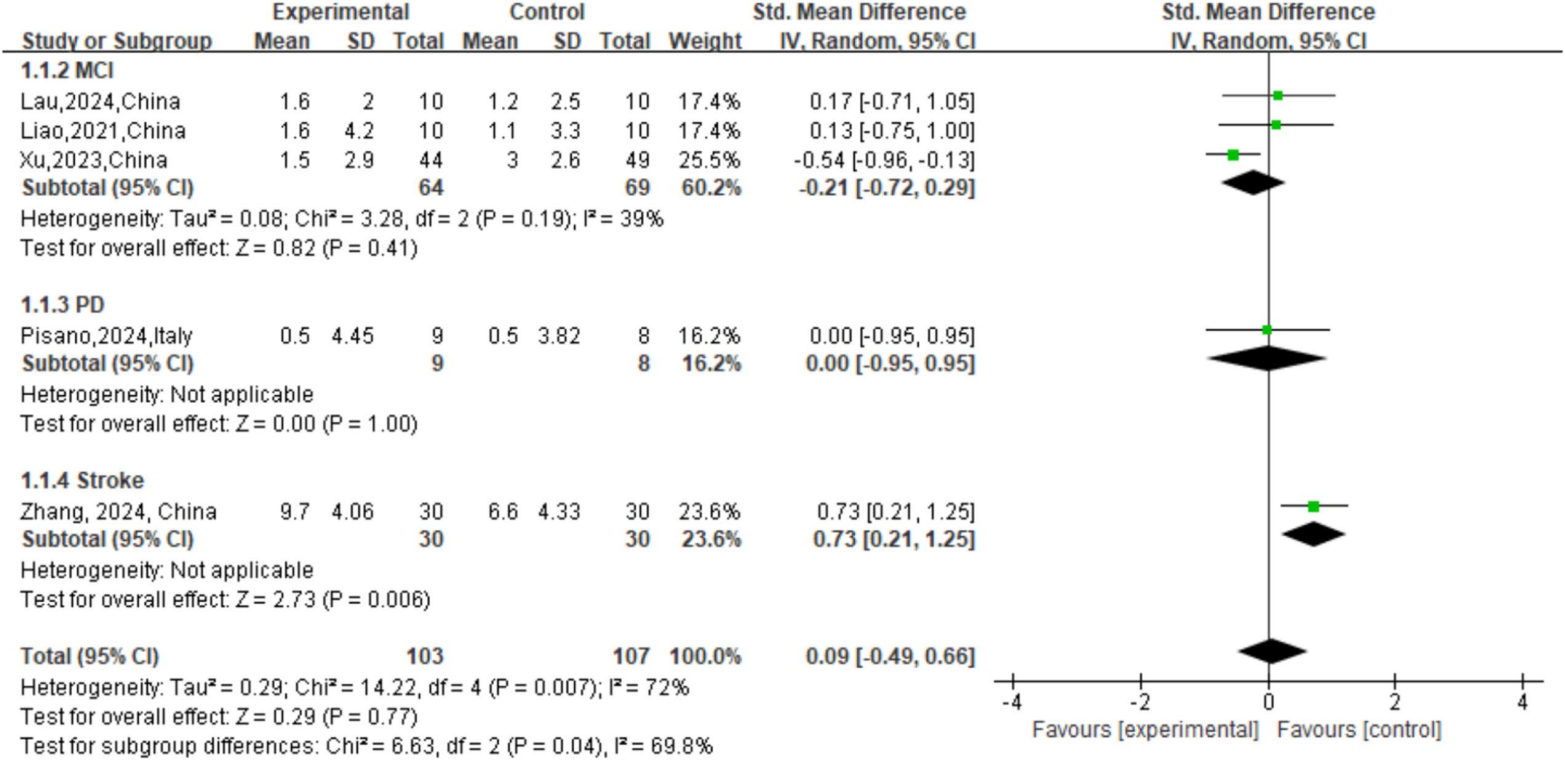
Forest plot of MoCA.

The SMD for the effect of active tDCS and sham group on MoCA score across all 5 studies was 0.09 (95% CI [−0.49, 0.66], *p* = 0.77). The analysis showed high statistical heterogeneity (I^2^ = 72%, *p* = 0.007), confirming significant variation in treatment effects across studies. A subgroup analysis based on the primary diagnosis (MCI, PD, Stroke) revealed a statistically significant difference between the subgroups ( $\chi^{2}$ =6.63, *p* = 0.04), suggesting that the effect of tDCS on MoCA scores is dependent on the patient population (Figure 4).

### 4.2 Secondary cognitive (TMT-B) outcome

**Figure 5:**
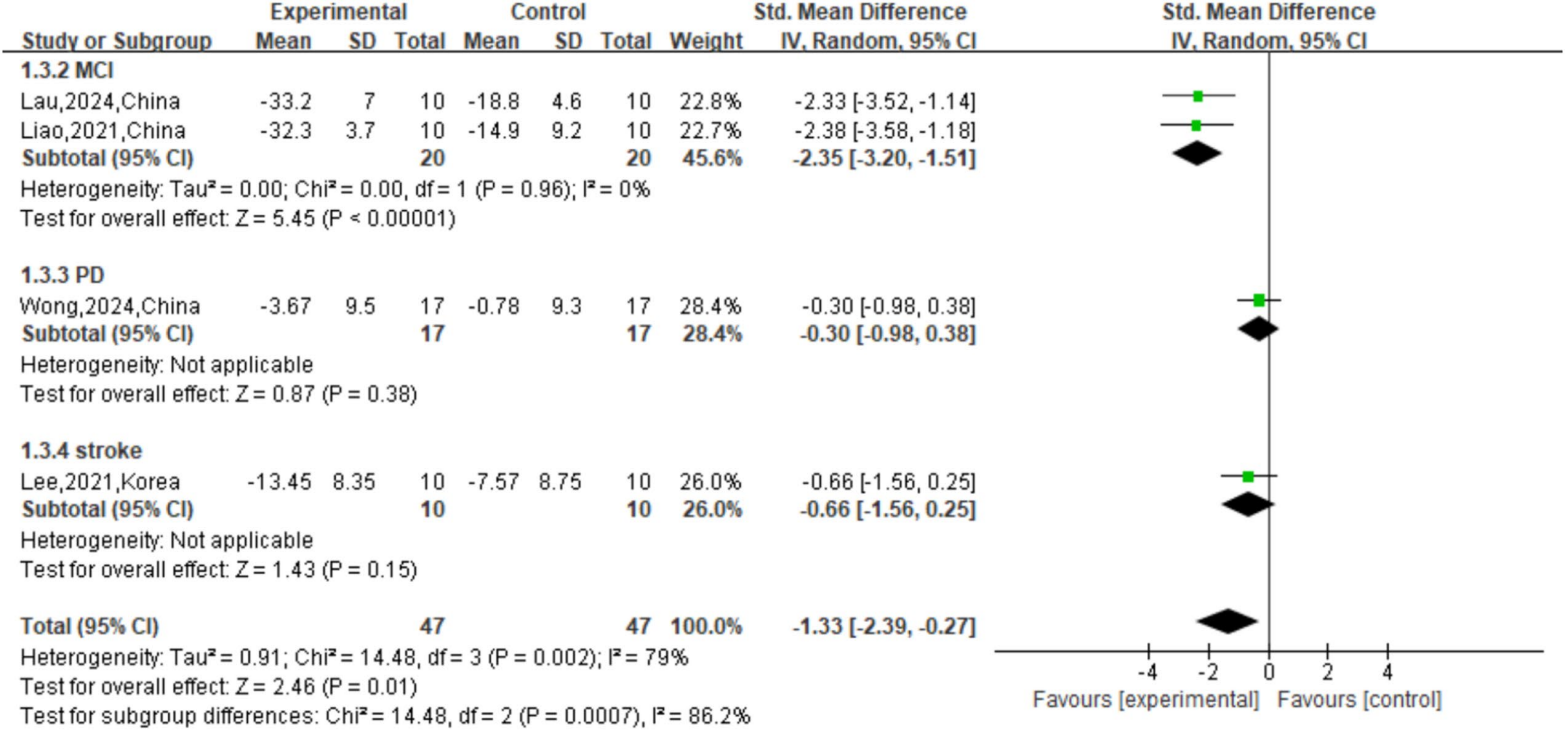
Forest plot of TMT-B.

Secondary cognitive outcome on TMT-B, meta-analysis revealed a large and significant overall benefit of active tDCS compared with sham (SMD = −1.33, 95% CI: [−2.39, −0.27], *p* = 0.01). However, substantial heterogeneity was observed across the overall sample (I^2^ = 79%, *p* = 0.002). Subgroup analysis demonstrated highly significant differences among groups ( $\chi^{2}$ =14.48, *p* < 0.001). The most pronounced and consistent effect was observed in patients with MCI, who showed a large, homogeneous, and highly significant improvement (SMD = −2.35, 95% CI: [−3.20, −1.51], I^2^ = 0%), reflecting substantial enhancement in executive function. Conversely, no significant benefits were observed in the PD or stroke subgroups (*p* > 0.05). These findings indicate that the efficacy of tDCS on this secondary cognitive domain is strongly condition-specific, with robust effects limited primarily to the MCI population (Figure 5).

### 4.3 Primary motor (TUG CMDT) outcome

**Figure 6:**
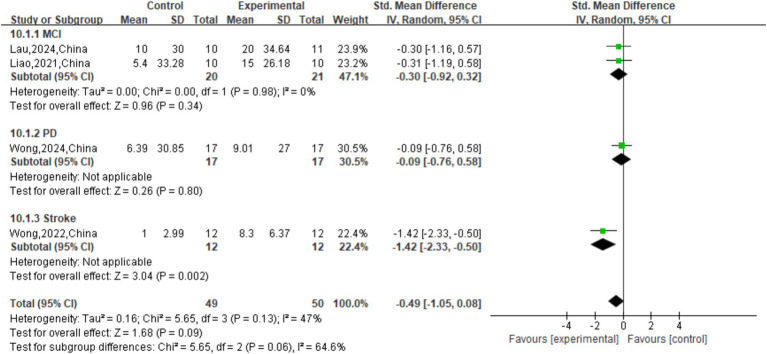
Forest plot of TUG CMDT speed (cm/s).

As for the primary motor outcome, TUG CMDT speed (cm/s), meta-analysis revealed a significant overall benefit favoring active tDCS (SMD = −0.49, 95% CI: [−1.05, 0.08], *p* = 0.09). Moderate heterogeneity was observed across the included studies (*p* = 0.13; I^2^ = 47%). Subgroup analysis by underlying disease showed no difference in treatment effect among groups ( $\chi^{2}$ =5.85, *p* = 0.06). Significant improvements were observed in the stroke subgroup (SMD = -1.42, 95% CI [−2.33, −0.50], *p* = 0.002). Conversely, in the MCI subgroup (2 studies) and the PD subgroup (1 study), the effects of the intervention were not statistically significant (MCI: SMD = -0.30, *p* = 0.34; PD: SMD = -0.09, *p* = 0.80; Figure 6).

### 4.4 Secondary motor [DTC, TUG ST, TUG motor DT, TUG CMDT cadence] outcomes

The effect of tDCS on TUG single speed was not significant (*p* = 0.50) compared to sham stimulation (SMD = 0.17 (95% CI: [−0.32, 0.66]); Figure 9). The overall effect of tDCS on DTC was not significant compared to sham stimulation (SMD = -0.29, 95% CI: [−0.73, 0.16], *p* = 0.21; Figure 10). As for cadence (steps/min) during the TUG CMDT (Figure 8), active tDCS showed a significant overall effect compared with sham (SMD = 0.58, 95% CI: [0.09, 1.08], *p* = 0.02), with low heterogeneity (I^2^ = 39%). Subgroup analysis revealed no significant difference between the three groups (χ^2^ = 2.67, *p* = 0.26). Significant improvements were observed in MCI (SMD = 0.93, 95% CI: [0.27, 1.58], *p* = 0.005) and stroke (SMD = 0.99, 95% CI: [0.13, 1.84], p = 0.02), whereas no significant benefit was observed in PD (SMD = 0.05, 95% CI: [−0.89, 0.99]). These findings suggest that tDCS selectively enhances CMDT cadence in MCI and stroke populations under cognitive-motor challenge (Figure 8).

**Figure 7:**
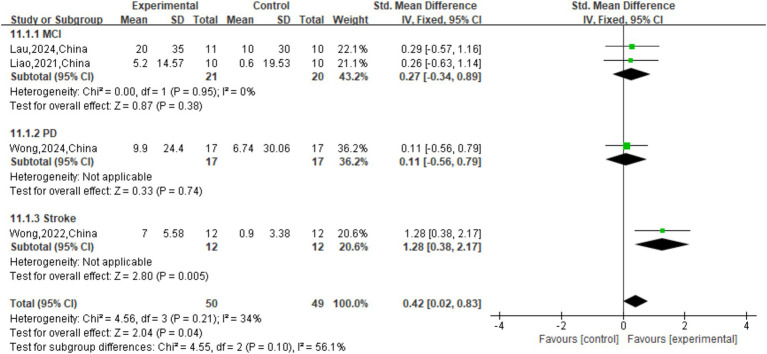
Forest plot of TUG motor dual task (cm/s).

**Figure 8:**
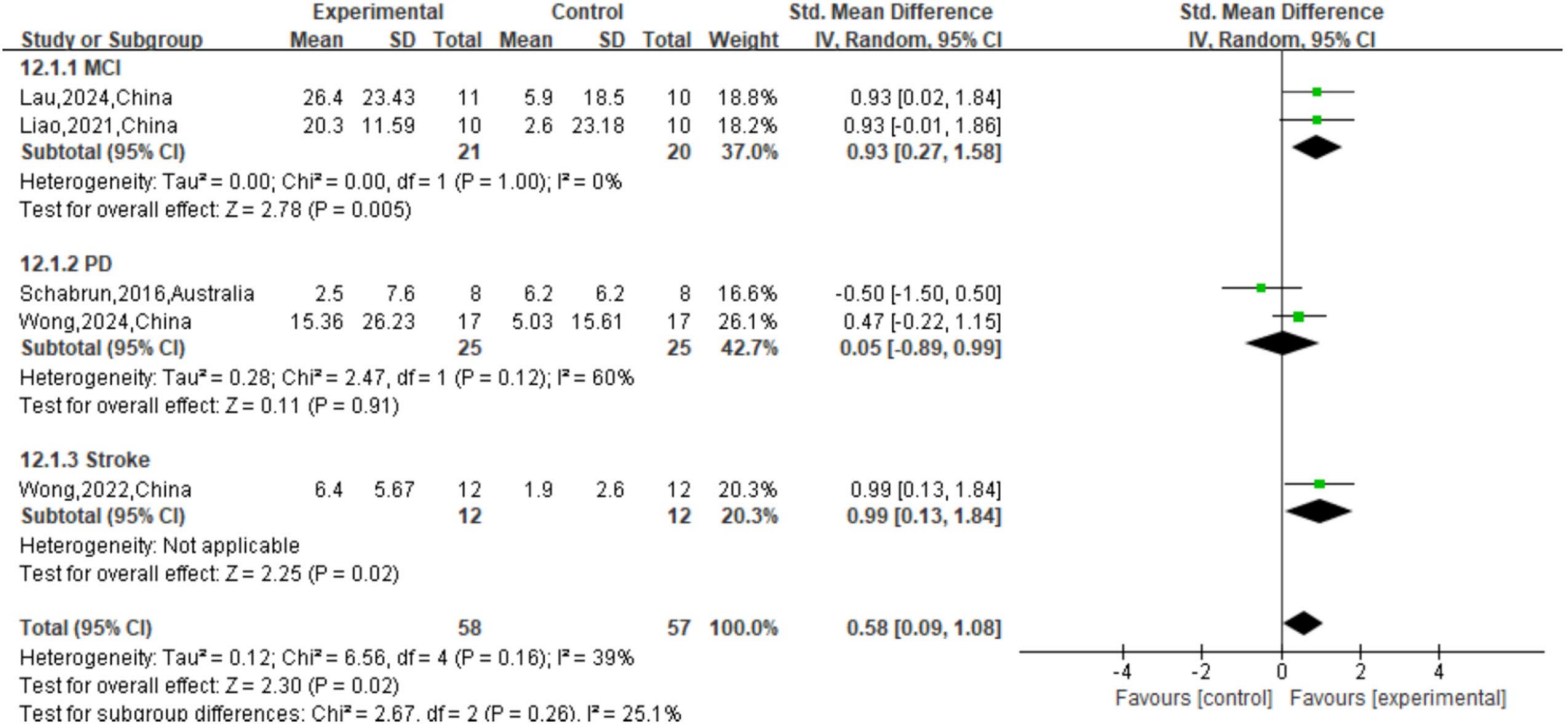
Forest plot of TUG CMDT cadence (cm/s).

**Figure 9:**
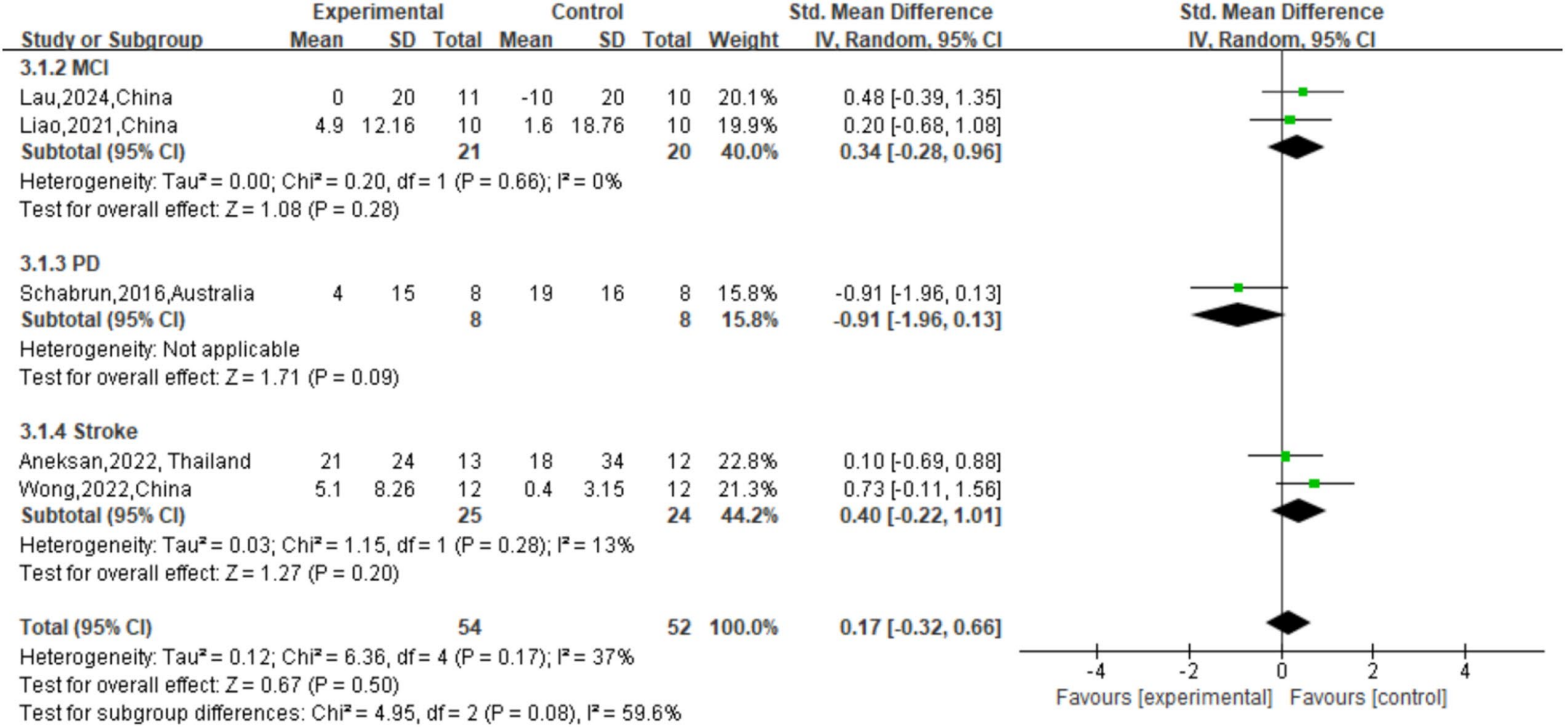
Forest plot of TUG single speed (cm/s).

**Figure 10:**
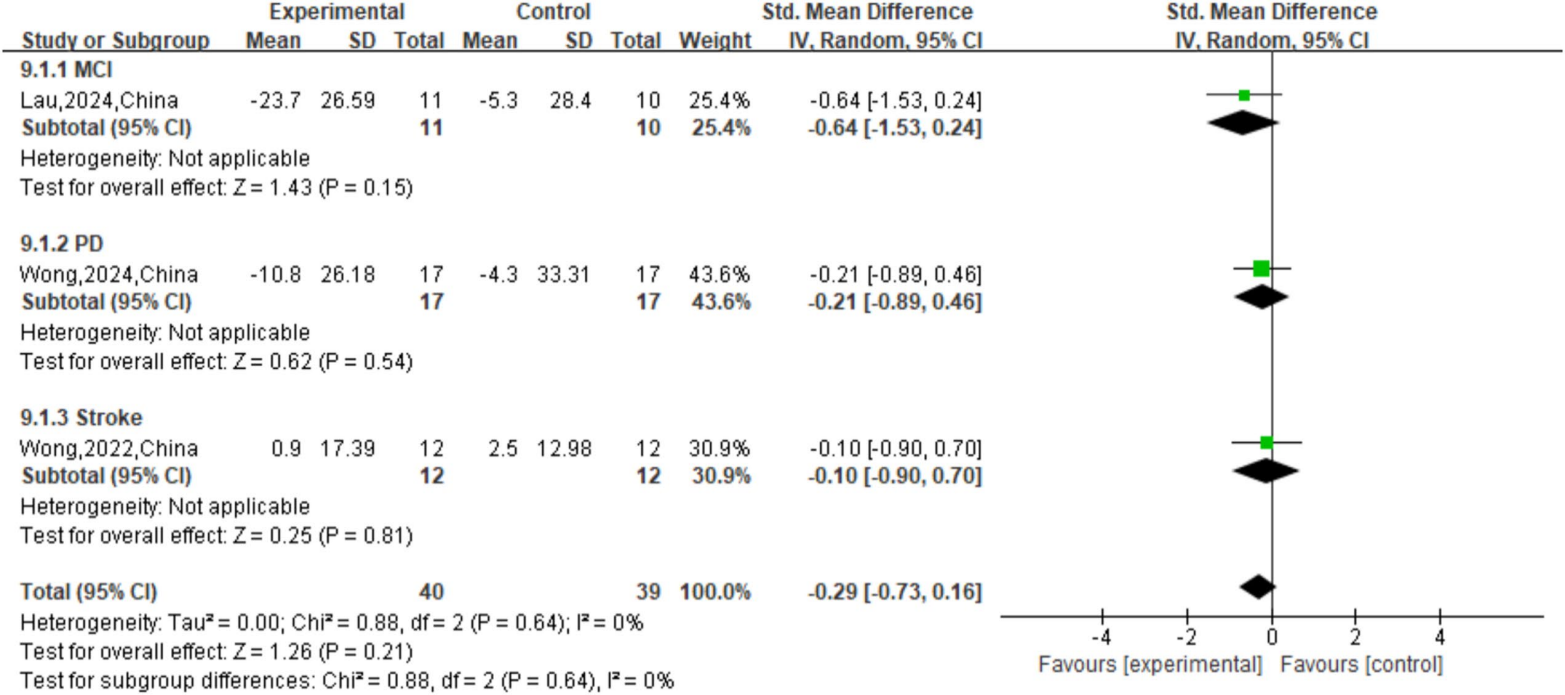
Forest plot of DTC.

There was a significant overall benefit of active tDCS compared to sham stimulation on TUG MDT speed (cm/s) with low heterogeneity (SMD = 0.42, 95% CI: [0.02, 0.83], p = 0.04, I^2^ = 34%; Figure 7). Figure 7 only the stroke subgroup showed significance (SMD = 1.28, 95% CI: [0.38, 2.17], p = 0.005). The effects observed in the MCI (SMD = 0.27, 95% CI: [−0.34, 0.89], *p* = 0.38) and PD (SMD = 0.11, 95% CI: [−0.56, 0.79], *p* = 0.74) subgroups were not significant. Importantly, the test for subgroup differences was non-significant ( $\chi^{2}$ =4.55, *p* = 0.10).

## 5 Discussion

This systematic review and meta-analysis represent the first comprehensive analysis specifically examining the combined effects of tDCS and DTT across three major neurological conditions (stroke, PD, and MCI). The results of this review revealed promising but inconsistent effects of tDCS on DT walking performance. Out of the 12 studies reviewed, five studies demonstrated significant improvements in CMDT cadence in the experimental groups compared to controls (Schabrun et al., 2016; Wong et al., 2022, 2024; Liao et al., 2021; Lau et al., 2024) and four studies revealed significant improvements in MDT speed (Wong et al., 2022, 2024; Liao et al., 2021; Lau et al., 2024), while four studies showed significant executive function (TMT-B) improvements (Liao et al., 2021; Lau et al., 2024; Wong et al., 2024; Xu et al., 2023). Two studies also reported significant increases in motor evoked potentials (MEP) in experimental groups (Wong et al., 2023, 2024). The intervention significantly improved tremor management (indicated by enhanced force-tremor decoupling; de Almeida et al., 2024) and generally enhanced postural stability (Pisano et al., 2024).

Meta-analysis revealed no overall improvement in global cognition, but demonstrated robust, highly homogeneous benefits in executive function (exclusively in MCI) and complex DT gait parameters (MCI and stroke). Conversely, conventional concurrent M1/left DLPFC tDCS showed no significant effects across all domains in PD. This apparent lack of efficacy in PD must be interpreted cautiously: included studies primarily targeted CMDT performance rather than core PD motor symptoms, and positive individual trials using cerebellar or Supplementary Motor Area (SMA/Cz) montages reported significant reductions in freezing of gait, postural instability, tremor, and force-tremor decoupling (Pisano et al., 2024; de Almeida et al., 2024). These findings underscore marked clinical and protocol heterogeneity.

Stroke participants derived the most pronounced and statistically robust benefits from tDCS combined with DTT. In the stroke subgroup, MDT speed improved significantly (SMD = 1.28, *p* = 0.005), CMDT cadence showed clear and large improvement (SMD = 0.99, *p* = 0.02), and CMDT speed demonstrated the largest effect size of all outcomes, specifically within the stroke subgroup (SMD = −1.42, *p* = 0.002).

These gains align with focal lesion pathophysiology, enabling neuroplastic reorganization (Chen et al., 2024), disrupting corticospinal/corticocortical tracts while preserving perilesional tissue (Gennaro et al., 2017). Anodal tDCS over ipsilesional M1/DLPFC boosts surviving neuron excitability and residual pathways, confirmed by increased MEP amplitudes (Edwards et al., 2009) and fMRI activation (Meinzer et al., 2016). DT gait impairment post-stroke results from automaticity loss and compensatory cognitive reliance (Hao et al., 2025), DLPFC/M1 targets are key executive nodes that facilitate cognitive-motor integration (Fu et al., 2025). Unlike PD’s dopaminergic depletion and basal ganglia-cortical disconnection impairing prefrontal modulation (Wong et al., 2024), stroke’s preserved plasticity results in direct gait enhancements. Effect sizes (7–12 cm/s DT speed increase) exceed minimal clinically important differences (0.10–0.16 m/s; Tilson et al., 2010), implying real-world mobility benefits.

The present systematic review and meta-analysis reveal that tDCS, when combined with CMDT or MDT, does not produce uniform benefits across major neurological disorders like MCI, PD, and stroke. Instead, its efficacy is highly domain-specific and condition-specific, with clinically meaningful effects confined to selected populations and functional domains. It provides critical insights into the effects of active tDCS on gait performance, particularly under DT conditions, compared to sham stimulation. While the overall effect on the primary motor outcome, TUG CMDT speed, showed a trend toward benefit (SMD = −0.49, *p* = 0.09), this finding was primarily driven by the robust and highly significant improvement observed in the stroke subgroup (SMD = −1.42, p = 0.002). This large effect suggests that tDCS effectively interacts with the motor and cognitive deficits inherent to stroke pathology, potentially by modulating cortical excitability in areas critical for gait and attention, thereby facilitating recovery and compensatory strategies (Sloane and Hamilton, 2024).

Specifically, tDCS produced a significant overall benefit for TUG CMDT cadence (SMD = 0.58, p = 0.02). This improvement was driven by significant gains in both MCI (SMD = 0.93, p = 0.005) and stroke (SMD = 0.99, p = 0.02) populations. This suggests that tDCS combined with DT training preferentially enhanced the rhythmic, attentional-control component of DT walking, which is captured by cadence, in people with MCI and stroke. Conversely, the non-significant effects on TUG single speed and DTC suggest that tDCS did not significantly influence single-task motor capacity or the overall cost of performing two tasks concurrently (Contemori et al., 2025).

There was a significant overall benefit observed for TUG MDT speed (SMD = 0.42, *p* = 0.04), again arising exclusively from the stroke subgroup (SMD = 1.28, p = 0.005), consistent with the CMDT speed findings. The consistency of these results across CMDT and MDT speed measures in stroke survivors strongly advocates for the inclusion of tDCS in stroke rehabilitation programs focused on functional mobility. The non-significant difference in subgroup effects for both TUG CMDT speed (*p* = 0.06) and TUG MDT speed (*p* = 0.10) further supports the conclusion that the intervention’s success is concentrated within the stroke cohort.

The pronounced efficacy observed in stroke survivors may be explained by the distinct neurobiological context of focal brain injury, which presents a clearer and more constrained window for neuroplastic reorganization (Chen et al., 2024). In contrast to the progressive and diffuse neurodegenerative changes seen in MCI and PD, stroke produces localized lesions that disrupt corticospinal and corticocortical pathways (Gennaro et al., 2017). Anodal tDCS applied over M1 or the DLPFC can enhance excitability in surviving perilesional neurons and strengthen remaining descending projections—an effect consistently demonstrated through increased MEP amplitudes (Edwards et al., 2009) and heightened ipsilesional activation on fMRI following stimulation (Yogev-Seligmann et al., 2008).

DT gait deficits after stroke are particularly pronounced due to the loss of gait automaticity and a compensatory shift toward cognitively mediated control mechanisms (Hao et al., 2025). Because both the DLPFC and M1 serve as central hubs within the executive control network that supports DT performance (Fu et al., 2025), their modulation via tDCS is more likely to yield meaningful functional gains in this population. Conversely, the extensive dopaminergic degeneration and basal ganglia–cortical disconnection characteristic of PD limit the effectiveness of prefrontal or motor-cortical stimulation (Wong et al., 2024; Filli et al., 2020). In summary, the preferential responsiveness of stroke survivors appears to arise from a combination of favorable neuroplastic potential, localized lesions with preserved perilesional circuitry, and a heightened dependence on corticospinal and prefrontal networks for compensatory gait control. Together, these factors create an optimal therapeutic landscape for M1- or DLPFC-targeted tDCS.

Notably, improvements were specific to DT contexts rather than single-task TUG or baseline gait parameters. This selective enhancement is theoretically consistent with cognitive resource models of DT performance in which mobility deficits arise from limited executive capacity and attentional allocation (Yogev-Seligmann et al., 2008; Filli et al., 2020). Anodal stimulation over the DLPFC or M1 likely enhances network efficiency within frontoparietal and motor circuits, improving the ability to coordinate cognitive processing and motor execution under simultaneous demands (Mishra and Thrasher, 2021). Complementing the mobility findings, executive function, as measured by using TMT-B, also showed a significant improvement (*p* = 0.01). TMT-B reflects cognitive flexibility, set-shifting, and inhibition, a key process for safe and adaptive DT walking. The parallel enhancement of TMT-B and cognitive DT gait performance supports a mechanistic interpretation. tDCS combined with DT may facilitate DT mobility by strengthening executive control networks that govern attentional switching and conflict monitoring (Mishra and Thrasher, 2021). This aligns with neurophysiological evidence showing that tDCS increases corticomotor excitability linked to cognitive control and enhances top-down modulation within the fronto-motor system (Koyun et al., 2025). The convergence of behavioral and electrophysiological findings supports a model in which tDCS promotes more efficient neural resource allocation, allowing patients to maintain gait performance even under cognitive load (Greeley et al., 2022).

Individual studies showed inconsistent effects across PD, stroke, and MCI populations (counting backwards by threes, naming animals; Peters et al., 2019), with some reporting significant improvements in cognitive DT walking performance (Wong et al., 2024), while others found no benefits beyond control interventions (Schabrun et al., 2016). This aligns with the central-peripheral-central closed-loop theory (Jia, 2022), emphasizing bidirectional brain-periphery feedback for plasticity (Bernhardt et al., 2020). Animal data link exercise to glial/neural coupling (Mandolesi et al., 2018), while tDCS activates limb function (Andre et al., 2016). DT performance integrates DLPFC (Nedović et al., 2024; Franchini et al., 2020), supplementary motor area/M1 (Hannah et al., 2018), and cerebellum (Wang et al., 2020), with aging disrupting gait-executive/attentional connectivity (Holtzer et al., 2014). Walking integrates sensory feedback, spinal networks, and descending control (Takakusaki, 2017). Post-stroke ipsilesional excitability loss disrupts interhemispheric balance, necessitating restoration for coordinated patterns (Hayes et al., 2023).

Additionally, in two studies (Wong et al., 2023, 2024), Motor Evoked Potentials (MEP) were used as a biomarker for functional recovery, with MEP cortical latency serving as a key indicator of motor cortex excitability (Lau et al., 2024). In stroke survivors, this latency is typically elevated due to structural and functional impairments in the affected cerebral hemisphere, making MEP valuable for assessing both corticospinal tract conduction and motor cortex excitability (Washabaugh et al., 2024). During rehabilitation, repeated motor cortex activation propagates to spinal motor neurons, creating automatic imprints that underlie automatic activity (Liao et al., 2022). The significant decrease in MEP cortical latency following combined tDCS and DTT in both Wong studies suggests that these interventions promote adaptive neural network changes consistent with neuroplasticity principles, effectively improving central physiological function and facilitating the reopening of damaged central conduction pathways. These findings align with observed neurophysiological changes, specifically increases in resting motor threshold in the stimulated hemisphere, suggesting that tDCS induces measurable neuroplastic changes even when functional improvements are not consistently detected across all outcome measures.

Another notable finding was the safety profile of tDCS interventions, with only two patients experiencing mild adverse reactions, accounting for just 2.2% of total cases (Wong et al., 2022). This low incidence aligns with the broader literature on tDCS, which consistently reports minimal side effects when administered according to established protocols. These findings support the conclusion that combined tDCS and DTT represent a safe intervention approach that can be implemented in clinical rehabilitation settings for patients with neurological conditions.

### 5.1 Limitation

The DT protocols varied considerably across the three populations with neurological disorders, preventing direct comparisons between pathologies. Similarly, tES parameters showed significant diversity in electrode placement, current intensity, and waveform characteristics, while treatment dosage ranged dramatically from a single session to 36 sessions, making it difficult to determine optimal protocols or establish dose–response relationships. A notable limitation is that, despite a comprehensive search strategy that included tES in its broadest sense—encompassing tDCS, tACS, and tRNS—only studies employing tDCS met the inclusion criteria for combined tES and DTT in MCI, PD, and stroke populations. No eligible trials utilizing tACS or tRNS in combination with DT gait or cognitive-motor training were identified during the search period up to November 2025. Consequently, the present findings and conclusions are specific to tDCS and cannot be generalized to other tES modalities. The absence of tACS and tRNS studies may reflect the relatively recent emergence of these techniques in cognitive-motor rehabilitation, their different hypothesized mechanisms, or simply a current evidence gap that future research should address.

## 6 Conclusion

This systematic review and meta-analysis provided the most comprehensive evidence to date on the effects of tDCS combined with DTT in MCI, PD, and stroke. The findings reveal markedly disease- and domain-specific efficacy rather than broad, cross-diagnostic benefits. Significant benefits were restricted to executive function (TMT-B). They selected DT gait parameters (CMDT cadence in MCI and stroke, MDT/CMDT speed in stroke only), with low-to-moderate heterogeneity (I^2^ 34–39%). The preferential efficacy in MCI and stroke reflects preserved perilesional/prefrontal substrates amenable to anodal M1/DLPFC-induced plasticity. Also, one PD trial demonstrated markedly increased CMDT accuracy with DLPFC stimulation. Emerging evidence from cerebellar and Cz-targeted tDCS suggests alternative montages may better address PD-specific deficits (postural instability, tremor, freezing of gait).

### 6.1 Clinical implications

tDCS+DTT is a promising adjunct for enhancing executive function and DT mobility in MCI and chronic stroke, but currently lacks support for routine use in PD with standard protocols.

### 6.2 Future directions

Large-scale RCTs with disease-tailored montages, standardized DTT, long-term follow-up, and direct comparison with tACS/tRNS are required to establish precision tES protocols and maximize translational impact across neurological populations.

## Data Availability

The datasets presented in this article are not readily available because data sharing not applicable to this article as no datasets were generated or analyzed during the current study. All other materials used in the review, including template data collection forms, analytic code, and additional review materials, are available within the article. Requests to access the datasets should be directed to Yutong Fu 906363326@qq.com.

## References

[ref1] Ali N. Tian H. Thabane L. Ma J. Wu H. Zhong Q. . (2022). The effects of dual-task training on cognitive and physical functions in older adults with cognitive impairment; a systematic review and Meta-analysis. J. Prev. Alzheimers Dis. 9, 359–370. doi: 10.14283/jpad.2022.16, 35543010

[ref2] Andre S. Heinrich S. Kayser F. Menzler K. Kesselring J. Khader P. H. . (2016). At-home tDCS of the left dorsolateral prefrontal cortex improves visual short-term memory in mild vascular dementia. J. Neurol. Sci. 369, 185–190. doi: 10.1016/j.jns.2016.07.065, 27653887

[ref3] Aneksan B. Sawatdipan M. Bovonsunthonchai S. Tretriluxana J. Vachalathiti R. Auvichayapat P. . (2022). Five-session dual-transcranial direct current stimulation with task-specific training does not improve gait and lower limb performance over training alone in subacute stroke: a pilot randomized controlled trial. Neuromodulation 25, 558–568. doi: 10.1111/ner.13526, 35667771

[ref5] Bernhardt J. Urimubenshi G. Gandhi D. B. C. Eng J. J. (2020). Stroke rehabilitation in low-income and middle-income countries: a call to action. Lancet 396, 1452–1462. doi: 10.1016/S0140-6736(20)31313-1, 33129396

[ref6] Blokland I. J. Schiphorst L. F. A. Stroek J. R. Groot F. P. van Bennekom C. A. M. van Dieen J. H. . (2023). Relative aerobic load of daily activities after stroke. Phys. Ther. 103:pzad005. doi: 10.1093/ptj/pzad005, 37172129 PMC10071588

[ref7] Brown R. E. Spratt T. J. Kaplan G. B. (2022). Translational approaches to influence sleep and arousal. Brain Res. Bull. 185, 140–161. doi: 10.1016/j.brainresbull.2022.05.002, 35550156 PMC9554922

[ref8] Chan M. M. Y. Yau S. S. Y. Han Y. M. Y. (2021). The neurobiology of prefrontal transcranial direct current stimulation (tDCS) in promoting brain plasticity: a systematic review and meta-analyses of human and rodent studies. Neurosci biobehav r 125, 392–416. doi: 10.1016/j.neubiorev.2021.02.035, 33662444

[ref9] Chen Y. Xu Z. Ma Y. Liu T. Tian X. Zhu Z. . (2024). Deep brain stimulation combined with morroniside promotes neural plasticity and motor functional recovery after ischemic stroke. Front. Pharmacol. 15:1457309. doi: 10.3389/fphar.2024.1457309, 39697542 PMC11652210

[ref10] Contemori G. Iorio C. Bonato M. (2025). The impact of dual-tasking on mnestic performance in normal ageing. Sci. Rep. 15:36131. doi: 10.1038/s41598-025-96784-z, 41102389 PMC12533128

[ref11] Cornea M. Vintilă B. I. Bucuța M. Ștef L. Anghel C. E. Grama A. M. . (2025). Efficacy of transcranial direct current stimulation and Photobiomodulation in improving cognitive abilities for Alzheimer's disease: a systematic review. J. Clin. Med. 14:1766. doi: 10.3390/jcm14051766, 40095881 PMC11900501

[ref12] Corominas-Teruel X. Bracco M. Fibla M. Segundo R. M. S. Villalobos-Llaó M. Gallea C. . (2023). High-density transcranial direct current stimulation to improve upper limb motor function following stroke: study protocol for a double-blind randomized clinical trial targeting prefrontal and/or cerebellar cognitive contributions to voluntary motion. Trials 24:783. doi: 10.1186/s13063-023-07680-8 38049806 PMC10694989

[ref13] de Almeida F. D. Wang Y. de Mello Pedreiro R. C. Brizzi A. C. B. Campos S. F. Sales M. P. . (2024). Combining transcranial direct current stimulation with exercise to improve mobility, stability, and tremor management in 25 individuals with Parkinson's disease. Neurol. Int. 16, 1223–1238. doi: 10.3390/neurolint16060093, 39585052 PMC11587078

[ref14] de Paz R. H. Serrano-Muñoz D. Pérez-Nombela S. Bravo-Esteban E. Avendaño-Coy J. Gómez-Soriano J. (2019). Combining transcranial direct-current stimulation with gait training in patients with neurological disorders: a systematic review. J. Neuroeng. Rehabil. 16:114. doi: 10.1186/s12984-019-0591-z 31521179 PMC6744683

[ref15] Ding X. T. Hu M. Y. Wang C. Kang W. Y. Huang J. Z. Wang R. Y. . (2025). The safety and effectiveness of tDCS for epileptic patients: a systematic review and meta-analysis. Complement. Ther. Med. 89:103142. doi: 10.1016/j.ctim.2025.103142, 39909364

[ref16] Duan Z. Zhang C. (2024). Transcranial direct current stimulation for Parkinson's disease: systematic review and meta-analysis of motor and cognitive effects. NPJ Parkinsons Dis 10:214. doi: 10.1038/s41531-024-00821-z 39505889 PMC11542032

[ref17] Edwards D. J. Krebs H. I. Rykman A. Zipse J. Thickbroom G. W. Mastaglia F. L. . (2009). Raised corticomotor excitability of M1 forearm area following anodal tDCS is sustained during robotic wrist therapy in chronic stroke. Restor. Neurol. Neurosci. 27, 199–207. doi: 10.3233/RNN-2009-0470, 19531875 PMC4510929

[ref18] Ehsani F. Jayedi A. Motaharinezhad F. Jaberzadeh S. (2025). The effects of transcranial direct current stimulation montages on motor learning across various brain regions: a systematic review and network meta-analysis. Neuroscience 569, 32–42. doi: 10.1016/j.neuroscience.2025.01.058, 39894438

[ref19] Filli L. Schwegler S. Meyer C. Killeen T. Easthope C. S. Broicher S. D. . (2020). Characterizing cognitive-motor impairments in patients with myotonic dystrophy type 1. Neuromuscul. Disord. 30, 510–520. doi: 10.1016/j.nmd.2020.04.005, 32527589

[ref20] Franchini E. Fukuda D. H. Lopes-Silva J. P. (2020). Tracking 25 years of judo results from the world championships and Olympic games: age and competitive achievement. J. Sports Sci. 38, 1531–1538. doi: 10.1080/02640414.2020.1747265, 32252597

[ref21] Fu Y. Yan Q. Wang A. Zhang H. Wang W. Yao L. (2025). Dual-target tDCS and dual-task training modulate neuroinflammation and neuroplasticity: transcriptomic and behavioral evidence in stroke rehabilitation. Front. Rehabil. Sci. 6. doi: 10.3389/fresc.2025.1589588, 41180413 PMC12575336

[ref22] Gaßner H. Trutt E. Seifferth S. Friedrich J. Zucker D. Salhani Z. . (2022). Treadmill training and physiotherapy similarly improve dual task gait performance: a randomized-controlled trial in Parkinson's disease. J. Neural Transm. (Vienna) 129, 1189–1200. doi: 10.1007/s00702-022-02514-4 35697942 PMC9463305

[ref23] Gennaro M. Mattiello A. Mazziotti R. Antonelli C. Gherardini L. Guzzetta A. . (2017). Focal stroke in the developing rat motor cortex induces age- and experience-dependent maladaptive plasticity of corticospinal system. Front. Neural Circ. 11:47. doi: 10.3389/fncir.2017.00047, 28706475 PMC5489564

[ref24] Giustiniani A. Maistrello L. Mologni V. Danesin L. Burgio F. (2025). TMS and tDCS as potential tools for the treatment of cognitive deficits in Parkinson's disease: a meta-analysis. Neurol. Sci. 46, 579–592. doi: 10.1007/s10072-024-07778-0, 39320648

[ref25] Greeley B. Barnhoorn J. S. Verwey W. B. Seidler R. D. (2022). Anodal transcranial direct current stimulation over prefrontal cortex slows sequence learning in older adults. Front. Hum. Neurosci. 16:814204. doi: 10.3389/fnhum.2022.814204, 35280208 PMC8907426

[ref26] Guerra A. Asci F. D'Onofrio V. Sveva V. Bologna M. Fabbrini G. . (2020). Enhancing gamma oscillations restores primary motor cortex plasticity in Parkinson's disease. J. Neurosci. 40, 4788–4796. doi: 10.1523/JNEUROSCI.0357-20.2020, 32430296 PMC7294804

[ref27] Han S. Luo J. Vo I. T. V. Chang W. K. Paik N. J. Choi J. S. . (2025). Motor system modulation by transcranial alternating current stimulation: insights from functional MRI-a scoping review. Front. Neurol. 16:1684725. doi: 10.3389/fneur.2025.1684725 41245871 PMC12615180

[ref28] Hannah R. Cavanagh S. E. Tremblay S. Simeoni S. Rothwell J. C. (2018). Selective suppression of local interneuron circuits in human motor cortex contributes to movement preparation. J. Neurosci. 38, 1264–1276. doi: 10.1523/JNEUROSCI.2869-17.2017, 29263237 PMC5792480

[ref29] Hao Y. Zhao Y. Luo H. Xie L. Hu H. Sun C. (2025). Comparative effectiveness of different dual task mode interventions on cognitive function in older adults with mild cognitive impairment or dementia: a systematic review and network meta-analysis. Aging Clin. Exp. Res. 37:139. doi: 10.1007/s40520-025-03016-5, 40304821 PMC12043736

[ref30] Hayes L. Taga M. Charalambous C. C. Raju S. Lin J. Schambra H. M. (2023). The distribution of transcallosal inhibition to upper extremity muscles is altered in chronic stroke. J. Neurol. Sci. 450:120688. doi: 10.1016/j.jns.2023.120688, 37224604 PMC10330477

[ref31] Higgins J. P. Altman D. G. Gøtzsche P. C. Jüni P. Moher D. Oxman A. D. . (2011). The Cochrane collaboration's tool for assessing risk of bias in randomised trials. BMJ 343:d5928. doi: 10.1136/bmj.d5928, 22008217 PMC3196245

[ref32] Hillel I. Gazit E. Nieuwboer A. Avanzino L. Rochester L. Cereatti A. . (2019). Is every-day walking in older adults more analogous to dual-task walking or to usual walking? Elucidating the gaps between gait performance in the lab and during 24/7 monitoring. Eur. Rev. Aging Phys. Act. 16:6. doi: 10.1186/s11556-019-0214-5 31073340 PMC6498572

[ref33] Hoh J. E. Semrau J. A. (2025). The role of sensory impairments on recovery and rehabilitation after stroke. Curr. Neurol. Neurosci. Rep. 25:22. doi: 10.1007/s11910-025-01407-9, 40047982 PMC11885399

[ref34] Holtzer R. Wang C. Verghese J. (2014). Performance variance on walking while talking tasks: theory, findings, and clinical implications. Age (Dordr.) 36, 373–381. doi: 10.1007/s11357-013-9570-7, 23943111 PMC3889876

[ref35] Jia J. (2022). Exploration on neurobiological mechanisms of the central-peripheral-central closed-loop rehabilitation. Front. Cell. Neurosci. 16:982881. doi: 10.3389/fncel.2022.982881, 36119128 PMC9479450

[ref36] Koyun A. H. Wendiggensen P. Roessner V. Beste C. Stock A. K. (2025). Neurophysiological insights into catecholamine-dependent tDCS modulation of cognitive control. Commun. Biol. 8:375. doi: 10.1038/s42003-025-07805-6, 40050533 PMC11885824

[ref37] Lau C. I. Liu M. N. Cheng F. Y. Wang H. C. Walsh V. Liao Y. Y. (2024). Can transcranial direct current stimulation combined with interactive computerized cognitive training boost cognition and gait performance in older adults with mild cognitive impairment? A randomized controlled trial. J. Neuroeng. Rehabil. 21:26. doi: 10.1186/s12984-024-01313-0, 38365761 PMC10874043

[ref38] Lee S. Cha H. (2022). The effect of clinical application of transcranial direct current stimulation combined with non-immersive virtual reality rehabilitation in stroke patients. Technol. Health Care 30, 117–127. doi: 10.3233/THC-212991, 34250916

[ref39] Lemke N. C. Werner C. Wiloth S. Oster P. Bauer J. M. Hauer K. (2019). Transferability and sustainability of motor-cognitive dual-task training in patients with dementia: a randomized controlled trial. Gerontology 65, 68–83. doi: 10.1159/000490852, 30041173

[ref40] Liao Y. Y. Liu M. N. Wang H. C. Walsh V. Lau C. I. (2021). Combining transcranial direct current stimulation with tai chi to improve dual-task gait performance in older adults with mild cognitive impairment: a randomized controlled trial. Front. Aging Neurosci. 13:766649. doi: 10.3389/fnagi.2021.766649, 34966268 PMC8710779

[ref41] Liao Y. T. Zheng Q. X. Huang P. P. Xie Q. L. Wang G. D. Lai Y. T. . (2022). Actual experience of the training effect of Baduanjin on patients with hemiplegic limb dysfunctions after cerebral infarction: a qualitative study. Nurs. Open 10, 861–868. doi: 10.1002/nop2.1354, 36161708 PMC9834197

[ref42] Longhurst J. K. Rider J. V. Cummings J. L. John S. E. Poston B. Landers M. R. (2023). Cognitive-motor dual-task interference in Alzheimer's disease, Parkinson's disease, and prodromal neurodegeneration: a scoping review. Gait Posture 105, 58–74. doi: 10.1016/j.gaitpost.2023.07.277, 37487365 PMC10720398

[ref43] Mandolesi L. Polverino A. Montuori S. Foti F. Ferraioli G. Sorrentino P. . (2018). Effects of physical exercise on cognitive functioning and wellbeing: biological and psychological benefits. Front. Psychol. 9:509. doi: 10.3389/fpsyg.2018.00509, 29755380 PMC5934999

[ref44] Martin S. S. Aday A. W. Almarzooq Z. I. Anderson C. A. M. Arora P. Avery C. L. . (2024). Heart disease and stroke statistics: a report of US and global data from the American Heart Association. Circulation 149, e347–e913. doi: 10.1161/CIR.0000000000001209, 38264914 PMC12146881

[ref45] Meinzer M. Darkow R. Lindenberg R. Flöel A. (2016). Electrical stimulation of the motor cortex enhances treatment outcome in post-stroke aphasia. Brain 139, 1152–1163. doi: 10.1093/brain/aww002, 26912641

[ref46] Mishra R. K. Thrasher A. T. (2021). Transcranial direct current stimulation of dorsolateral prefrontal cortex improves dual-task gait performance in patients with Parkinson's disease: a double blind, sham-controlled study. Gait Posture 84, 11–16. doi: 10.1016/j.gaitpost.2020.11.012, 33260076

[ref47] Morelli N. Summers R. L. S. (2023). Association of subthalamic beta frequency sub-bands to symptom severity in patients with Parkinson's disease: a systematic review. Parkinsonism Relat. Disord. 110:105364. doi: 10.1016/j.parkreldis.2023.105364 36997437

[ref48] Nedović N. Eminović F. Marković V. Stanković I. Radovanović S. (2024). Gait characteristics during dual-task walking in elderly subjects of different ages. Brain Sci. 14. doi: 10.3390/brainsci14020148, 38391723 PMC10886897

[ref49] Oh E. Park J. Youn J. Jang W. (2022). Anodal transcranial direct current stimulation could modulate cortical excitability and the central cholinergic system in Akinetic rigid-type Parkinson's disease: pilot study. Front. Neurol. 13:830976. doi: 10.3389/fneur.2022.830976, 35401397 PMC8987019

[ref50] Page M. J. McKenzie J. E. Bossuyt P. M. Boutron I. Hoffmann T. C. Mulrow C. D. . (2021). The PRISMA 2020 statement: an updated guideline for reporting systematic reviews. BMJ 372:n71. doi: 10.1136/bmj.n71, 33782057 PMC8005924

[ref51] Peristeri E. Vogelzang M. Tsimpli I. M. (2021). Bilingualism effects on the cognitive flexibility of autistic children: evidence from verbal dual-task paradigms. Neurobiol. Lang. (Camb) 2, 558–585. doi: 10.1162/nol_a_00055, 37214625 PMC10198706

[ref52] Peters S. Eng J. J. Liu-Ambrose T. Borich M. R. Dao E. Amanian A. . (2019). Brain activity associated with dual-task performance of ankle motor control during cognitive challenge. Brain Behav. 9:e01349. doi: 10.1002/brb3.1349, 31265216 PMC6710191

[ref53] Pisano F. Mellace D. Fugatti A. Aiello E. N. Diotti S. Curti B. . (2024). Cerebellar tDCS combined with augmented reality treadmill for freezing of gait in Parkinson's disease: a randomized controlled trial. J. Neuroeng. Rehabil. 21:173. doi: 10.1186/s12984-024-01457-z, 39342307 PMC11438075

[ref54] Qin Y. Xu J. Ng S. S. M. (2025). Effects of transcranial direct current stimulation (tDCS) on motor function among people with stroke: evidence mapping. Syst. Rev. 14:60. doi: 10.1186/s13643-025-02795-2, 40069897 PMC11899689

[ref55] Sánchez-Kuhn A. Pérez-Fernández C. Cánovas R. Flores P. Sánchez-Santed F. (2017). Transcranial direct current stimulation as a motor neurorehabilitation tool: an empirical review. Biomed. Eng. Online 16:76. doi: 10.1186/s12938-017-0361-8 28830433 PMC5568608

[ref56] Schabrun S. M. Lamont R. M. Brauer S. G. (2016). Transcranial direct current stimulation to enhance dual-task gait training in Parkinson's disease: a pilot RCT. PLoS One 11:e0158497. doi: 10.1371/journal.pone.0158497, 27359338 PMC4928827

[ref57] Sethi A. Pascual-Leone A. Santarnecchi E. Almalki G. Krishnan C. (2023). Transcranial random noise stimulation to augment hand function in individuals with moderate-to-severe stroke: a pilot randomized clinical trial. Restor. Neurol. Neurosci. 41, 193–202. doi: 10.3233/RNN-231314, 38306067

[ref58] Sloane K. L. Hamilton R. H. (2024). Transcranial direct current stimulation to ameliorate post-stroke cognitive impairment. Brain Sci. 14:614. doi: 10.3390/brainsci14060614, 38928614 PMC11202055

[ref59] Steen-García L. Franco-Jiménez R. Ibáñez-Alfonso J. A. (2024). Transcranial direct current stimulation (tDCS) in adults with attention deficit hyperactivity disorder. A systematic review. Rev. Neurol. 79, 239–246. doi: 10.33588/rn.7909.2024294, 39540387 PMC11605908

[ref100] Steinmetz J. D. Seeher K. M. Schiess N. Nichols E. Cao B. Servili C. . (2024). Global, regional, and national burden of disorders affecting the nervous system, 1990–2021: a systematic analysis for the Global Burden of Disease Study 2021. The Lancet Neurology, 23, 344–381. doi: 10.1016/S1474-4422(24)00038-3, 38493795 PMC10949203

[ref60] Takakusaki K. (2017). Functional neuroanatomy for posture and gait control. J Mov Disord 10, 1–17. doi: 10.14802/jmd.16062, 28122432 PMC5288669

[ref61] Takeuchi N. Izumi S. I. (2021). Motor learning based on oscillatory brain activity using transcranial alternating current stimulation: a review. Brain Sci. 11:1095. doi: 10.3390/brainsci11081095, 34439714 PMC8392205

[ref62] Tilson J. K. Sullivan K. J. Cen S. Y. Rose D. K. Koradia C. H. Azen S. P. . (2010). Meaningful gait speed improvement during the first 60 days poststroke: minimal clinically important difference. Phys. Ther. 90, 196–208. doi: 10.2522/ptj.20090079, 20022995 PMC2816032

[ref63] Trombini-Souza F. de Maio Nascimento M. da Silva T. F. A. de Araújo R. C. Perracini M. R. Sacco I. C. N. (2020). Dual-task training with progression from variable- to fixed-priority instructions versus dual-task training with variable-priority on gait speed in community-dwelling older adults: a protocol for a randomized controlled trial: variable- and fixed-priority dual-task for older adults. BMC Geriatr. 20:76. doi: 10.1186/s12877-020-1479-2 32087694 PMC7036177

[ref64] Tuena C. Borghesi F. Bruni F. Cavedoni S. Maestri S. Riva G. . (2023). Technology-assisted cognitive motor dual-task rehabilitation in chronic age-related conditions: systematic review. J. Med. Internet Res. 25:e44484. doi: 10.2196/44484 37213200 PMC10242476

[ref65] van der Groen O. Potok W. Wenderoth N. Edwards G. Mattingley J. B. Edwards D. (2022). Using noise for the better: the effects of transcranial random noise stimulation on the brain and behavior. Neurosci biobehav r 138:104702. doi: 10.1016/j.neubiorev.2022.104702 35595071

[ref66] van der Groen O. Wenderoth N. (2016). Transcranial random noise stimulation of visual cortex: stochastic resonance enhances central mechanisms of perception. J. Neurosci. 36, 5289–5298. doi: 10.1523/JNEUROSCI.4519-15.2016, 27170126 PMC6601807

[ref67] Wang L. Yu Y. Ying S. Wang M. Qin Y. Liu T. . (2025). A personalized closed-loop brain stimulation protocol for difficulty falling asleep. IEEE Trans. Neural Syst. Rehabil. Eng. 33, 2368–2380. doi: 10.1109/TNSRE.2025.3572851, 40408188

[ref68] Wang H. Zhang W. Zhao W. Wang K. Wang Z. Wang L. . (2020). The efficacy of transcranial alternating current stimulation for treating post-stroke depression: study protocol clinical trial (SPIRIT compliant). Medicine (Baltimore) 99:e19671. doi: 10.1097/MD.0000000000019671, 32311940 PMC7220515

[ref69] Washabaugh E. P. Foley S. A. Czopek E. G. Krishnan C. (2024). Altered corticospinal and intracortical excitability after stroke: a systematic review with meta-analysis. Neurorehabil. Neural Repair 38, 845–862. doi: 10.1177/15459683241281299, 39275953

[ref70] Wiltshire C. E. E. Watkins K. E. (2020). Failure of tDCS to modulate motor excitability and speech motor learning. Neuropsychologia 146:107568. doi: 10.1016/j.neuropsychologia.2020.107568 32687836 PMC7534039

[ref71] Wong P. L. Yang Y. R. Huang S. F. Wang R. Y. (2023). Effects of transcranial direct current stimulation followed by treadmill training on dual-task walking and cortical activity in chronic stroke: a double-blinded randomized controlled trial. J. Rehabil. Med. 55:jrm00379. doi: 10.2340/jrm.v55.5258, 36943024 PMC10065121

[ref72] Wong P. L. Yang Y. R. Huang S. F. Wang R. Y. (2024). Effects of DLPFC tDCS followed by treadmill training on dual-task gait and cortical excitability in Parkinson's disease: a randomized controlled trial. Neurorehabil. Neural Repair 38, 680–692. doi: 10.1177/15459683241268583, 39104216

[ref73] Wong P. L. Yang Y. R. Tang S. C. Huang S. F. Wang R. Y. (2022). Comparing different montages of transcranial direct current stimulation on dual-task walking and cortical activity in chronic stroke: double-blinded randomized controlled trial. BMC Neurol. 22:119. doi: 10.1186/s12883-022-02644-y, 35337288 PMC8951706

[ref74] Wu X. L. Lu S. X. Wang X. X. Dong G. Q. Lu M. Y. Zhang Z. H. . (2025). Effect of ultrasound-guided acupotomy combined with acupuncture on limb dysfunction in patients with cerebral stroke. Neurol. Sci. 46, 2707–2716. doi: 10.1007/s10072-025-08072-3, 40048119 PMC12084177

[ref75] Xu Y. Zhu J. Liu H. Qiu Z. Wu M. Liu J. . (2023). Effects of tai chi combined with tDCS on cognitive function in patients with MCI: a randomized controlled trial. Front. Public Health 11:1199246. doi: 10.3389/fpubh.2023.1199246, 37608981 PMC10441111

[ref76] Yao P. Zhou Q. Ren B. Yang L. Bai Y. Feng Z. (2025). Transcranial pulsed current stimulation alleviates neuronal pyroptosis and neurological dysfunction following traumatic brain injury via the orexin-a/NLRP3 pathway. Neuropeptides 110:102501. doi: 10.1016/j.npep.2025.102501, 39764896

[ref77] Yogev-Seligmann G. Hausdorff J. M. Giladi N. (2008). The role of executive function and attention in gait. Mov. Disord. 23, 329–342, 18058946 10.1002/mds.21720 PMC2535903

[ref78] Zegarra-Valdivia J. A. Shany-Ur T. Rijpma M. G. Callahan P. Poorzand P. Grossman S. . (2025). Validation of the cognitive-emotional perspective taking test in patients with neurodegeneration. J Alzheimer's Dis 104, 436–451. doi: 10.1177/13872877251317683, 40026013 PMC12231792

[ref79] Zhang L. Ma J. Liu X. Jin A. Wang K. Yin X. (2024b). Cognitive-motor dual-task training on gait and balance in stroke patients: meta-analytic report and trial sequential analysis of randomized clinical trials. J. Neuroeng. Rehabil. 21:227. doi: 10.1186/s12984-024-01507-6 39716165 PMC11665123

[ref80] Zhang X. Xu F. Shi H. Liu R. Wan X. (2022). Effects of dual-task training on gait and balance in stroke patients: a meta-analysis. Clin. Rehabil. 36, 1186–1198. doi: 10.1177/02692155221097033, 35469457

[ref81] Zhang L. Zhou L. Ye Q. Zhang L. Kong Y. Xia S. (2024a). Impact of transcranial direct current stimulation combined with motor-cognitive intervention on post-stroke cognitive impairment. Neurol. Sci. 45, 1581–1588. doi: 10.1007/s10072-023-07156-2, 37923844

